# Gaussian regressor-based adaptive control of exoskeleton joints in the presence of system uncertainty

**DOI:** 10.1017/wtc.2025.9

**Published:** 2025-08-26

**Authors:** Mohamed Abdelhady, Thomas C. Bulea

**Affiliations:** Rehabilitation Medicine Department, National Institutes of Health Clinical Center, Bethesda, MD, USA

**Keywords:** adaptive control, exoskeleton, gait, wearable robotics

## Abstract

System uncertainty remains a challenge for effective control of lower extremity exoskeletons, particularly in clinical populations. Adaptive control offers a potential solution by accounting for unknown system characteristics in real time. Here, we introduce the use of Gaussian-based adaptive control (GBAC) in a two-degree-of-freedom (DOF) exoskeleton for an angular position tracking task in the presence of system uncertainty. The mathematical derivation of the implicitly non-Lyapunov adaptation law is presented using Lagrangian mechanics, including a Gaussian kernel regressor and its stable convergence. We then evaluate GBAC performance in a 2-DOF simulation compared with a previously developed robust adaptive backstepping algorithm, Lyapunov-stable Slotine–Li control, and a proportional-integral-derivative (PID) controller. We additionally complete 1-DOF simulations to evaluate the effects of external disturbance and parameter uncertainty on controller performance. Finally, we evaluate GBAC experimentally in our existing 1-DOF knee exoskeleton along with Slotine–Li and PID controllers. The simulation results demonstrate the improved tracking performance and faster convergence of GBAC, especially in the presence of an external disturbance and uncertainty introduced by extra segment length and mass. The experimental results demonstrate similar performance, wherein GBAC and Slotine–Li provide stable tracking in the presence of unmodeled system dynamics; however, convergence time was faster and tracking error was lower for GBAC. Collectively, these results demonstrate that GBAC is an effective adaptive controller in the presence of system uncertainty and therefore warrants further development and investigation for use in flexible joint exoskeleton systems, particularly those designed for pediatric and/or clinical populations that have inherently high uncertainty.

## 1. Introduction

Lower extremity robotic exoskeletons have emerged as transformative tools for addressing neuromotor disorders such as stroke, spinal cord injury, and cerebral palsy, as well as for enhancing mobility in healthy individuals (Chang et al., 2018; Nam et al., 2019; Yamamoto et al., 2022). As the exoskeleton user population has expanded, so have their operational objectives (Mooney et al., 2014). For individuals with paralysis, exoskeletons serve as assistive devices to restore lost ambulation capability (Tran et al., 2021). In those with partial mobility, they function as both assistive tools to facilitate walking and rehabilitation aids to improve volitional movement (Lerner et al., 2017; Bulea et al., 2022). In healthy individuals, exoskeletons augment physical performance by reducing the energy cost of walking or running and enhancing load-carrying capacity (Sawicki et al., 2020). A common goal across these applications is the transition of exoskeleton use from controlled clinical or laboratory settings to real-world environments. To achieve this objective, the development of robust and adaptable control systems capable of operating reliably under diverse conditions is crucial (Rupal et al., 2017).

From a mechanical and control perspective, exoskeleton joints are inherently flexible due to their attachment to human limbs (Young and Ferris, 2016; Zhu et al., 2016; Lee et al., 2017; Rupal et al., 2017). This flexibility introduces significant challenges, including vibration, oscillation, friction, backlash, and other nonlinearities, which can compromise tracking accuracy and system stability (Yang et al., 2020). Additionally, flexible joints are sensitive to environmental and user-induced disturbances, such as variations in load or joint stiffness, further complicating control efforts (Bartenbach et al., 2016; Aliman et al., 2018). Exoskeletons are also subject to a wide range of uncertainties, including external disturbances (e.g., uneven terrain or unexpected collisions) and parametric variations (e.g., changes in limb size, mass, or inertia). Many uncertainties are particularly pronounced in clinical populations, including unmodeled dynamics (e.g., reflexive or velocity-dependent spastic muscle responses, as well as unexpected muscle activation patterns and forces), day-to-day variance in muscle function correlated with impairment severity, and atypical biomechanical properties (e.g., altered muscle and bone structures), which can significantly impact system performance.

To address these challenges, advanced control strategies that combine modeling, feedback control, and feedforward compensation have been developed to mitigate vibrations, nonlinearities, and time delays. Exoskeleton control systems typically employ a hierarchical architecture, where a high-level controller governs system behavior and a low-level controller implements desired joint torques or movements in real time. A critical area of research involves integrating low-level adaptation laws with high-level controllers to dynamically adjust exoskeleton goals during operation (Wang et al., 2016; Narayan et al., 2023). Adaptive control is particularly attractive for exoskeletons due to its ability to account for system uncertainties and update parameters in real time, enabling operation in unpredictable environments and during activity transitions (Slade et al., 2022). Adaptive approaches can be broadly categorized as either model-based or model-free. Whereas model-based methods excel in controlled settings, their performance can degrade in the presence of unmodeled dynamics, external disturbances, parameterization errors, and measurement inaccuracies (Meng et al., 2022; Liu et al., 2023; Masengo et al., 2023). Model-free approaches can circumvent these limitations but require substantial training data, are sensitive to initial conditions, and often lack interpretability, complicating optimization and troubleshooting.

Significant progress has been made in developing adaptive control systems for lower-limb exoskeletons. Zhang et al. (2018) combined neural networks (NNs) with time delay control to achieve superior trajectory tracking compared with traditional proportional-derivative (PD) control. Zhu et al. (2016) employed radial basis NNs to enhance tracking accuracy and reduce interaction forces. Han et al. (2018) developed a model-free adaptive controller integrating time-delay estimation and adaptive sliding mode control, demonstrating stability via Lyapunov theory. Other notable contributions include adaptive feedforward NNs for compliant systems (Asl et al., 2018), recurrent NNs for reliable force and position tracking (Zhang et al., 2019), and adaptive robust integral of sign error controllers for asymptotic stability under disturbances (Sherwani et al., 2020).

Real-time adaptive control is especially promising for clinical populations, such as stroke and cerebral palsy, where unpredictable muscle activation due to spasticity and volitional impairment poses unique challenges. Adaptive control has been successfully implemented in single-joint exoskeletons, such as those providing proportional ankle assistance or scaled knee extension support (Gasparri et al., 2019; Chen et al., 2021). Maalej et al. (2019) further advanced this field by combining extended 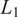 adaptive control with PID controllers to achieve accurate trajectory tracking in 2-degree-of-freedom (DOF) exoskeletons in the presence of system uncertainties, demonstrating effectiveness in simulation across a range of limb masses and lengths as would be expected in children.

Despite these advancements, system uncertainties, particularly in clinical populations, remain a significant hurdle. Among these, unmodeled dynamics represent a critical challenge, as they can lead to significant tracking errors and instability, especially in individuals with neuromotor disorders. Adaptive exoskeleton control must account for this uncertainty in the system dynamics, and Gaussian kernels are well suited to do so because they are bounded, and linear combinations of Gaussians can approximate any continuous function. Inspired by prior work on these Gaussian kernel functions for online learning (Petrič, 2020) and radial basis NNs, we propose a hybrid adaptive control approach for lower-limb exoskeletons that combines a Lagrangian mechanics-based dynamical model, which eliminates the need for a large training dataset to learn the system dynamics, with a model-free adaptation law based on Gaussian kernels, which we term Gaussian-based adaptive control (GBAC). GBAC offers a computationally efficient and inherently stable adaptive control paradigm capable of implicitly accounting for disturbances and uncertainties. The primary objectives of this study are to implement the GBAC control rule, evaluate its ability to capture system dynamics, and quantify its tracking and convergence performance in simulation and benchtop experiments. Specifically, GBAC is evaluated in 1- and 2-DOF simulations in the presence of external disturbances and parametric uncertainties, and experimentally in 1-DOF testing with unmodeled dynamics/external disturbance in the form of a spring and parametric uncertainty in the form of unexpected additional mass. Collectively, these uncertainties are commonly experienced in clinical populations and represent a significant barrier to the implementation of accurate trajectory tracking in these individuals. Stability analysis is out of the scope of this paper and is reserved for future work.

The remainder of this paper is organized as follows: Section 2 provides the mathematical modeling of flexible joint systems. Section 3 describes the derivation of the flexible joint GBAC. Section 4 evaluates GBAC against existing adaptive and traditional control methods in 1- and 2-DOF exoskeleton simulations as well as a 1-DOF experimental knee joint trajectory tracking task. Finally, Section 5 discusses the results and outlines future research directions.

## 2. System modeling

**Figure 1.**
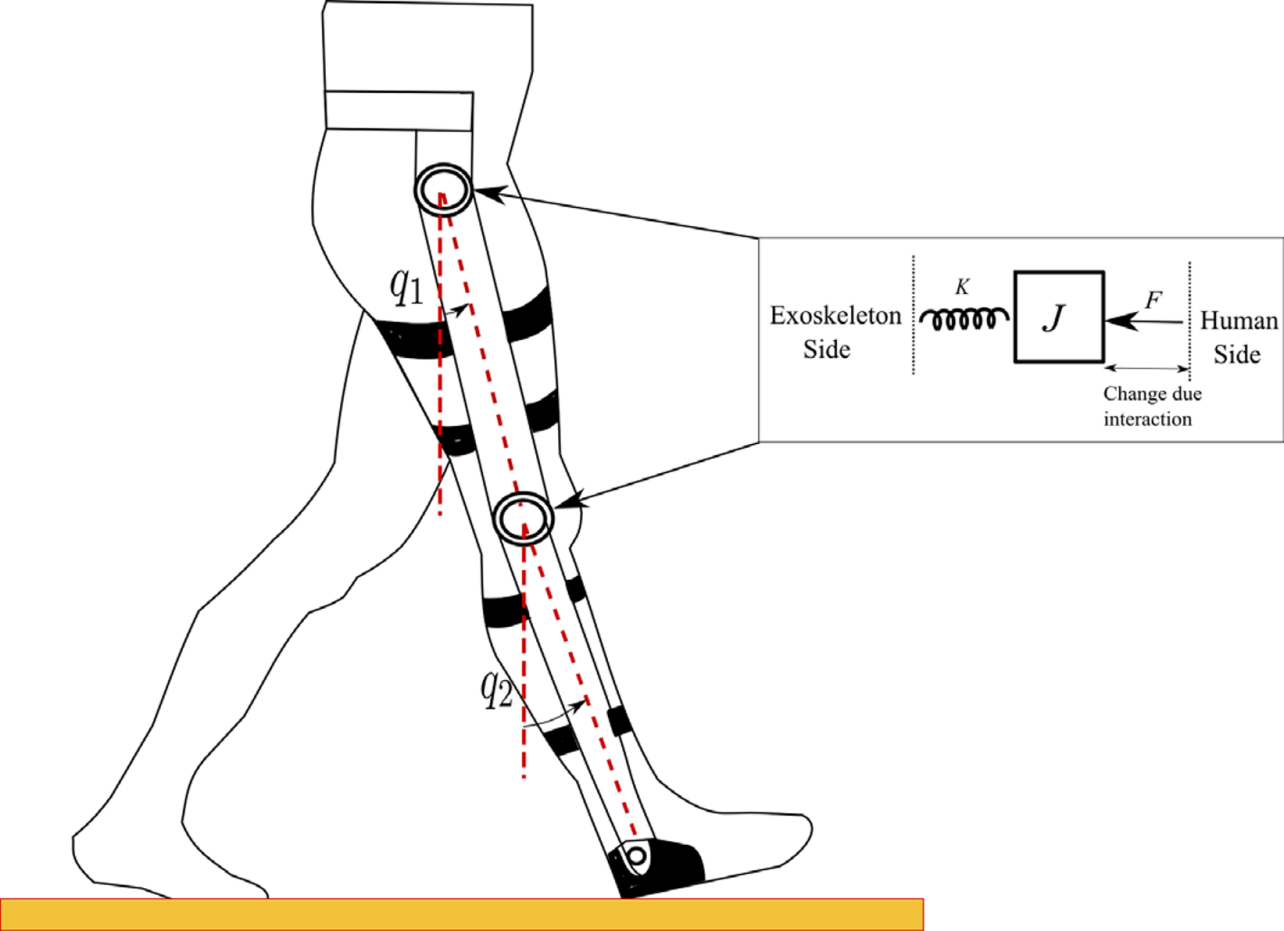
Biomechanical model of an exoskeleton-assisted walking system in the sagittal plane, showing the exoskeleton’s structure and its interaction with human joints. The dynamic model uses the hip ( 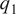 ) and knee ( 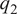 ) joint angles as generalized coordinates to illustrate the relationship among exoskeleton angular position, joint stiffness, damping, and human joint behavior.

We utilize the Lagrangian method, which is based on energy analysis, to find the dynamic equation of the model. Each leg of the lower-limb exoskeleton is represented by a 2-DOF mechanism, in which its hip and knee joints are flexibly actuated in the sagittal plane as shown in Figure 1. The uncertainty of each flexible actuated joint is modeled by the displacement between the flexible joint and any point on the rigid frame as(1)

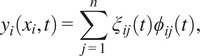

where 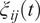 is time-dependent coordinates of the joint and 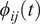 is the spatially dependent 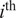 mode shape function of link 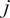 (Rahimi and Nazemizadeh, 2014; Shalaby, 2018). In our exoskeleton model, the links are fixed-mass beams that are flexible in two directions within the sagittal plane, that is, each has two modes of vibration. Their mode shapes were defined using their boundary conditions. The mode shapes were normalized to a magnitude of 1 (i.e., unit vector) to simplify the final model.

For the case of two modes of vibration for each link, the kinetic and potential energy expressions for the two joints, the two links, and the links’ mass were derived using the kinematics relationships between the segments. The final equations of motion were then derived using Lagrange energy techniques and coincided with those presented by De Luca et al. (1991). The model of the system is represented as follows:(2)

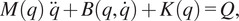

where 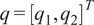 is the generalized coordinate vector, 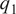 and 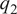 are the hip and knee angle (Figure 1), 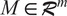 is the inertia matrix, 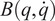 is the vector of Coriolis centrifugal forces and the motor and structural damping terms, 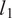 is the stiffness matrix, and 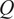 is the vector of generalized forces.

In this work, we assume that the flexible links move in a plane where the gravitational effect exists, and the flexible links are rigidly coupled to two actuated joints. The exoskeleton utilizes brushless DC (BLDC) motors to actuate the joints that are modeled as(3)

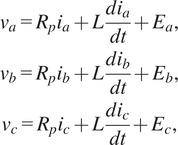

where *a*, *b*, and *c* are motor windings, 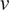 is the phase voltage (V), 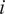 is the phase current (A), 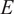 is the back electromotive force (EMF), 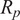 is the phase resistance in ( 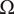 ), and 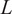 is the phase inductance (H). The ultimate BLDC motor torque, derived from the previous equation, is as in Lund et al. (2019):(4)

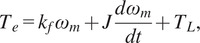

where 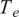 is the generated electrical torque (Nm), 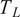 is the mechanical load, 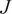 is the rotor inertia (kg m^2^), 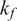 is the frictional constant (Nm/rad), and 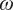 is the rotor speed (rad/s).

For each joint, equation (4) estimates the joint torques in the Lagrange formulation of the exoskeleton equations of motion, considering 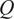 in equation (2) as(5)

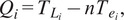

where 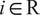 is the number of exoskeleton revolute joints.

## 3. Adaptive control design

To derive our adaptive control law, we utilized a previously developed analytical model of a two-link, two-actuator system, assuming clamped boundary conditions and two vibration modes per link as in Mutti et al. (1998) and Zhang et al. (2005).

The goal of the adaptive control law is to precisely control the joint angles to track the desired trajectories in the presence of system uncertainty. Taking into account the system uncertainties produced by imprecise modeling, such as system parameter perturbation, external disturbances, and non-ideal boundary conditions, the dynamic equations of the two flexible link system, as formulated in equation (3), can be rewritten as(6)

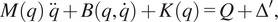

where 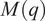 is the inertia matrix, 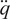 is the acceleration vector, 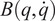 is the Coriolis and centripetal forces, 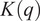 is the gravitational forces, 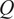 is the generalized forces, and 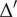 is the uncertainties vector with its components being continuous functions of 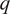 and 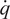 , encompassing modeling errors, external perturbations, and unmodeled dynamics. The essential properties of equation (6) are defined as

**Property (1):** The inertial matrix 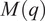 is a symmetric positive-definite matrix and satisfies 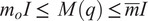 , where 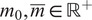 and 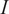 is the identify matrix;

**Property (2):** The matrix 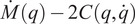 is skew-symmetric such that 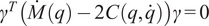 , where 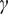 is any real vector such that 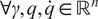 ;

**Property (3):** The dynamics of the links are linearly parameterized such that 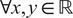 , 

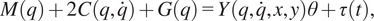

where 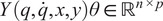 is a regressor matrix and 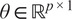 are constants with high uncertainty, such as exoskeleton physical parameters like link mass, moments of inertia, or other dynamic properties. Collectively, this term represents joint displacement due to uncertainty, as in equation (1). Additionally, 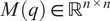 is the positive-definite inertia matrix, 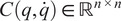 is the Coriolis and centripetal torque matrix, 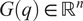 denotes the gravitational force, and 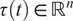 denotes the control torque. We can then reformulate equation (6) by decomposing into separate rigid and flexible modes:(7)

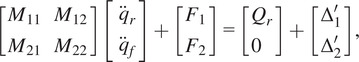

where 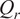 is the vector of intrinsic and external rigid torques, 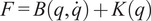 , rigid angle vector 
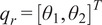
 , and flexible angle vector 
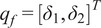
 . From equation (7), we can define(8)

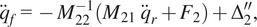

where 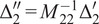 . Substituting (8) into the lower row of (7), it follows that(9)

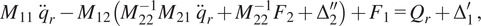


(10)

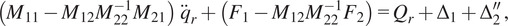


(11)

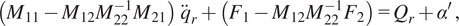

where 
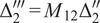
 and 
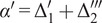
 are the vectors quantifying system uncertainty as unknown continuous functions of 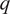 and 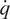 . Because 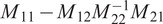 is nonsingular everywhere in motion space, 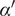 can be written as(12)

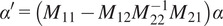



Let 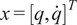 and 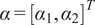 . Because 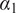 and 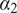 are continuous functions in 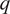 and 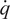 , 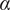 can be approximated using a Gaussian regressor (more details can be found in Sanner and Slotine, 1992) with 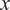 being the input vector, such as(13)

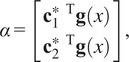

where 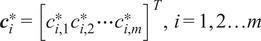 , are the output weight vectors, 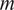 is the number of nodes in the Gaussian regressor, and the Gaussian basis function vector is selected as(14)

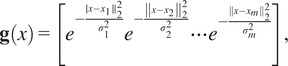

where 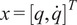 and 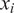 and 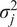 , 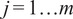 , are the center points and the variances, respectively. Using equation (7) to compute joint torques 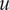 , we let(15)

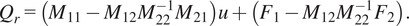



The joint torques 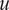 can then be calculated as(16)

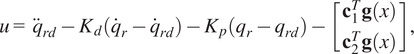

where 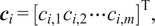 and the joint index and Gaussian node index are 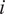 and 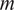 , respectively. If the desired joint angle is 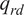 , the system dynamics with 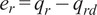 and 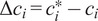 become(17)

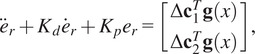


(18)

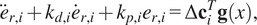

where 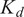 and 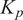 are selected as diagonal matrices. To achieve stable performance, the feedback gains of equation (17) are selected with negative real roots, such as(19)

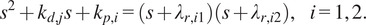



If we consider the update mechanism output as(20)

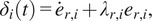

then we can show that(21)

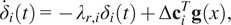

where 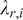 is selected to achieve a reasonable convergence 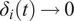 or 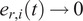 as 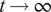 .

The adaptive law for this system can then be formulated as(22)

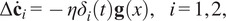

where 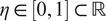 is the adaptation gain that governs the rate of the adaptation process. Additional information regarding the asymptotic stability of this update law can be found in the Appendix. In order to compute the torque vector needed to track a desired trajectory based on the Lagrangian dynamic equation of motion, a fixed number of Gaussian nodes is selected to form the adaptation law. In this work, we utilized five nodes. For additional details on the Gaussian node structure, please refer to Sanner and Slotine (1992). The update law is calculated by local integration to correct the computed torque values iteratively as in equation (16). We call this control scheme a Gaussian-based adaptive control (GBAC).

## 4. Control assessment

Here, we utilized a simulation evaluation to compare the performance of our novel proposed GBAC control with a robust adaptive backstepping (BS) controller that adaptively updates the unknown dynamic exoskeleton parameters derived from Lagrange mechanics, and was previously deployed in a trajectory tracking task for a lower extremity exoskeleton (Narayan et al., 2023). We also implemented a well-known adaptive control scheme derived based on Lyapunov stability (Slotine–Li; Slotine and Weiping, 1988) and a PID controller as baseline comparators. The simulation study evaluates algorithm tracking and convergence for a range of exoskeleton user limb masses and lengths, which represent practical sources of parametric uncertainty in pediatric populations who experience periods of rapid growth and development. In this work, we utilized our existing 1-DOF knee exoskeleton mechanism (Chen et al., 2021) to experimentally evaluate and compare the novel GBAC, Slotine–Li, and PID controllers during a trajectory tracking task. The experimental evaluation provides a focused application of controller performance in the presence of unmodeled system dynamics induced by a spring representing a possible change in user muscle tone or spasticity as well as the addition of limb mass.

### 4.1. Simulation study

#### 4.1.1. 2-DOF exoskeleton system

We first focus on a lower-limb exoskeleton with 2-DOF at the hip and knee joint in the sagittal plane, where each has flexible joint capability (Figure 1). The inertia, Coriolis matrix, and gravitational vector for the exoskeleton’s equations of motion (equation (3)) are defined as(23)

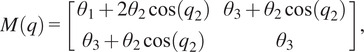


(24)

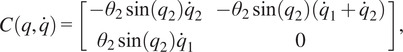


(25)

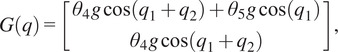

where 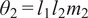 , 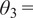 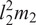 , 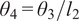 , 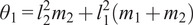 , 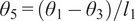 , and the masses of the exoskeleton links are chosen as 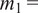 2 kg, 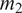 = 0.5 kg, 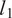 = 0.35 m, and 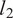 = 0.25 m, which are based on our existing exoskeleton hardware. The desired trajectory is set as 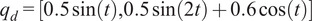 . The simulation study implements the proposed GBAC control update law (equation (22)) and compares its performance with the well-known Slotine–Li adaptive controller (Huang and Chien, 2010; Abdelhady and Simon, 2020) since it has a stable adaptation based on Lyapunov stability. All the tuning procedures for the Slotine–Li controller were performed as described in Abdelhady and Simon (2020). The PID controller was selected as a baseline controller. The following assumptions are made during the simulation:

1. The desired joint trajectory 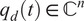 is continuous, bounded differentiable up to the fourth order.

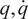
 and 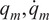 are measurable during 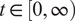 .
3. All unmodeled nonlinear dynamics, such as backlash and friction, are considered in the velocity motion profile.
4. The hip and knee trajectories imply the existence of the concept of persistent excitation.

The simulation study considers two cases. In the first, the update laws for GBAC and Slotine–Li controllers were calculated with mass and link lengths near to the model values. The second case considers controller behavior in a high uncertainty situation, that is, where all the exoskeleton linkage weights and lengths are 100% above the nominal values of the mechanical model. Because the PID controller does not have an adaptation law, the PID gains were first tuned using link lengths and weights 5% above the nominal values of the original mathematical model. PID performance was then tested twice, at the model values and with model parameters 100% above the original model. The PID gains were tuned using the MATLAB control system toolbox and optimization toolbox. Hip and knee PID controller gains are [33.6, 19.8, and 10.7] and [40, 12, 27] for P, I, and D gains, respectively. The robust adaptive BS controller was implemented as in Narayan et al. (2023). The Slotine–Li controller adaptation law derivation was described in Abdelhady and Simon (2020).

To simulate the proposed adaptive control scheme, we identified five Gaussian regressor nodes for each joint (equation (13)). The initial value of each node is 0, and the initial values of 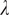 and 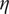 are selected using trial and error to be [10 10] and 100, respectively. Next, the MATLAB single-step solver (ode23) was utilized to solve equation (22). All simulations were carried out with a sampling time of 10 ms. The root mean square (RMS) error from the reference trajectory was computed for the first 2 s of the simulation to evaluate the controller’s performance and convergence.

**Figure 2.**
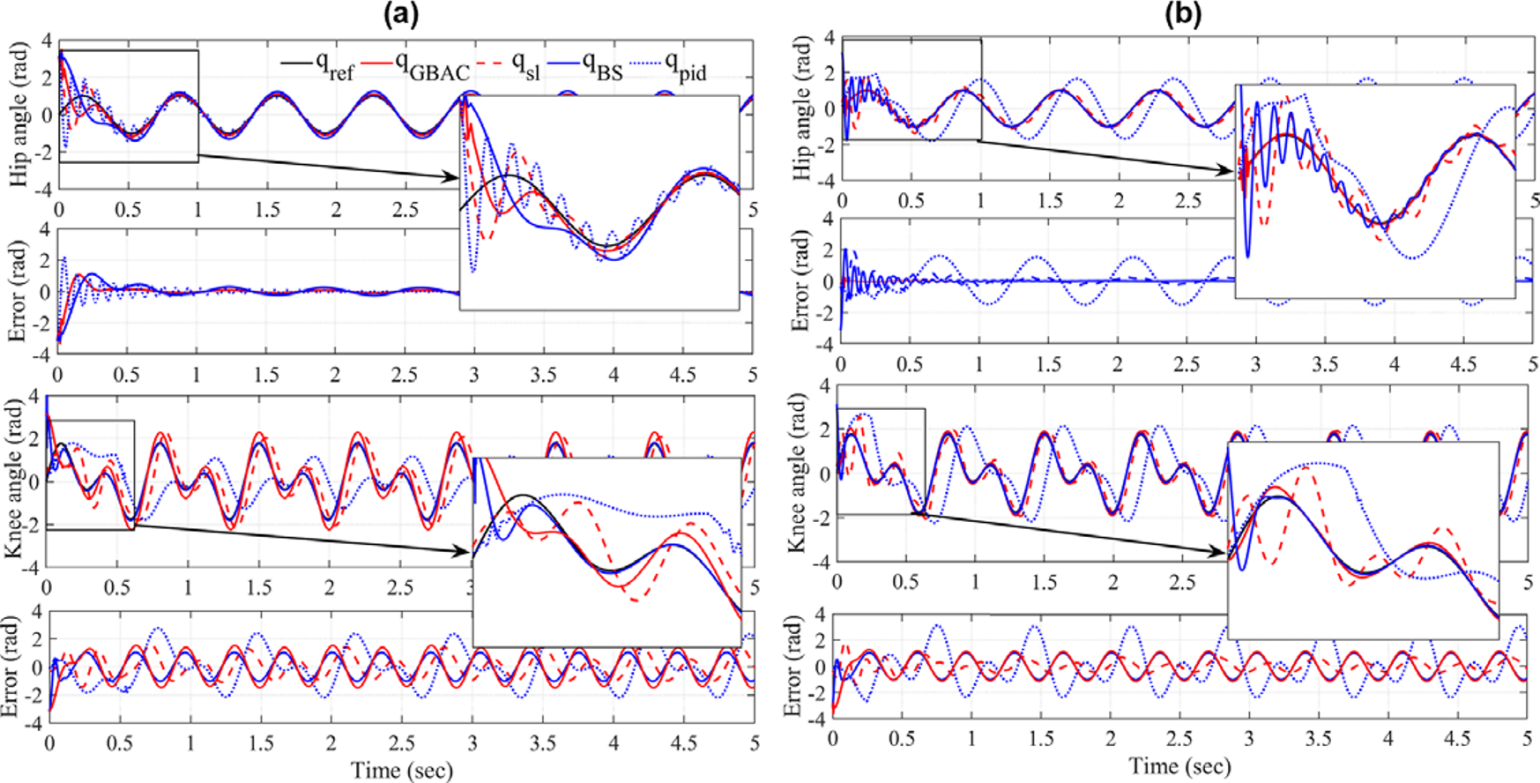
(a) Controller performance with model parameters for link mass and length 5% above the mathematical model at the hip (top) and the knee (bottom) indicated by joint angles and error from reference trajectory for Gaussian-based adaptive control ( 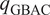 ), Slotine–Li ( 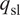 ), BS ( 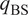 ), and PID ( 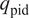 ), respectively. The insets show controller convergence in the first two cycles. (b) Controller performance presented in the same format as (a) but with model parameters for link mass and length 100% above those in the mathematical model.

When the model parameters were close to their expected levels, the tracking performance of all controllers was similarly effective, with convergence in <0.6 s and RMS error <0.4 rad (Figure 2). Notably, GBAC had the fastest convergence (<0.2 s). The performance contrast between controllers was more apparent at the knee. GBAC and BS converged faster than the Slotine–Li controller, with BS converging slightly faster and with a slightly lower RMS (0.66 rad) compared with GBAC (0.76 rad), Slotine–Li (0.86 rad), and PID (1.1 rad). This improved performance can be attributed to the GBAC and BS adaptation mechanisms, which are based on regression and robustness, rather than the Lyapunov-based stability adaptation deployed in Slotine–Li. The PID controller provides relatively good tracking at the hip, whereas at the knee PID controller error was the highest due to the uncontrolled coupling between the two joints.

As expected, the PID controller had the worst tracking performance due to high uncertainty from the extra mass and length, and the lack of adaptability to handle the coupling effect (Figure 2b). At the hip, GBAC, BS, and Slotine–Li converge to less than 0.5-rad RMS error; however, GBAC converged faster than Slotine–Li and BS. At the knee, Slotine–Li shows poorer tacking performance (RMS = 0.47 rad) compared with BS (0.41 rad) and GBAC (0.35 rad), which performed the best. GBAC also converged slightly faster than BS in this case.

#### 4.1.2. 1-DOF exoskeleton system

To evaluate the performance of controllers under a wider range of disturbances and uncertainties and facilitate a comparison with our existing exoskeleton utilized in the experimental study, a 1-DOF simulation was completed. The 1-DOF exoskeleton system is modeled as a single pendulum with a torsional spring at the joint, viscous friction, and an external disturbance force 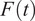 . The system dynamics are governed by the following equation of motion:(26)

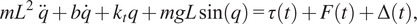

where 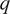 is the angular displacement of the exoskeleton, 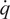 and 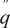 are the angular velocity and acceleration, respectively, 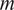 is the mass of the exoskeleton’s pendulum, 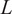 is the length of the pendulum, 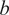 is the viscous damping coefficient, 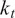 is the torsional spring constant, 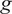 is the acceleration due to gravity, 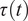 is the control input torque applied at the joint, 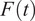 is the external disturbance force, and 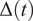 represents the uncertainty in the system parameters. The system is initialized at 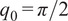 rad, and the controllers aim to track a reference trajectory defined as(27)

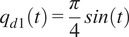

while rejecting the disturbance 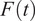 and compensating for the uncertainty 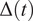 .

**Figure 3.**
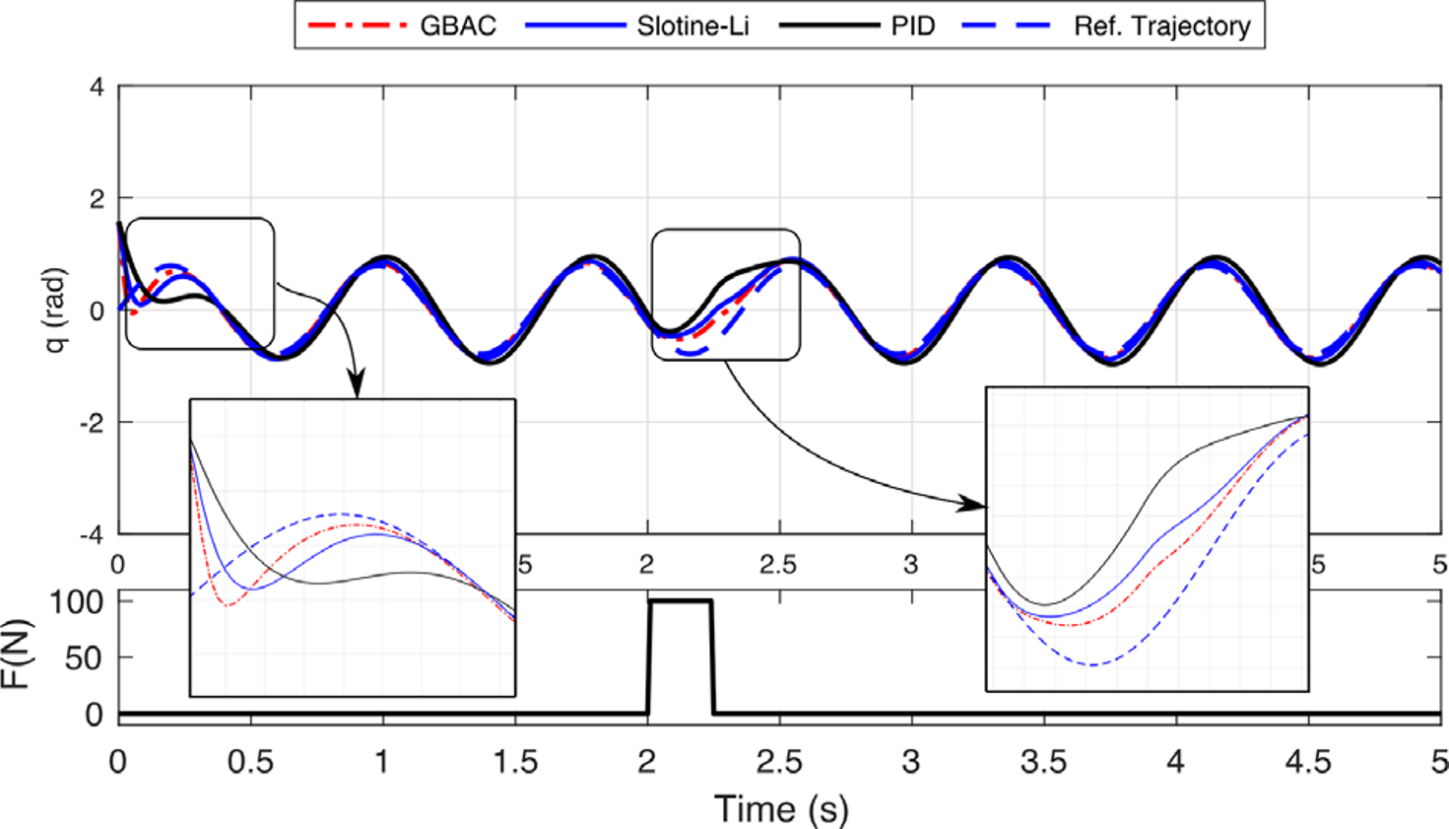
Simulation results comparing the performance of controllers in response to a 100 N external disturbance force 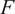 applied between 2 and 2.25 s. The system starts from the initial conditions at 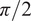 rad. The plots show the simulated reference trajectory 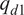 and the system’s tracking response under GBAC, Slotine–Li, and PID controllers. The insets show the initial convergence and effect of the applied disturbance and recovery dynamics.

We first evaluated the ability of three previously described controllers – GBAC, Slotine–Li, and PID – to handle a 100 N external disturbance force 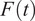 applied briefly between 2 and 2.25 s. The disturbance is modeled as(28)

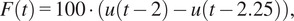

where 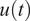 is the unit step function. As shown in Figure 3, the GBAC controller, implemented with five Gaussian nodes, demonstrated superior performance in maintaining trajectory tracking despite the disturbance, compared with the Slotine–Li and PID controllers, with an RMS error of 0.27 rad across the simulation window (5 s). The PID controller, with gains 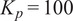 , 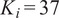 , and 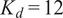 , exhibited a slower response and larger error during the disturbance period, resulting in the largest RMS (0.44 rad). The Slotine–Li controller exhibited performance closer to the GBAC controller, but with a higher RMS error of 0.35 rad.

Finally, we examined the behavior of the GBAC under parameter uncertainties in the 1-DOF exoskeleton. The initial condition is set at 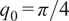 rad, with the exoskeleton tasked to follow a sinusoidal reference trajectory. The nominal parameters of the exoskeleton were selected to match operational values for human applications, including the frequency of the sinusoidal trajectory and the physical parameters of the exoskeleton system. The simulation was then completed to evaluate the GBAC tracking performance of the exoskeleton joint angle 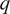 in the presence of a set of unexpected parameter changes.

**Figure 4.**
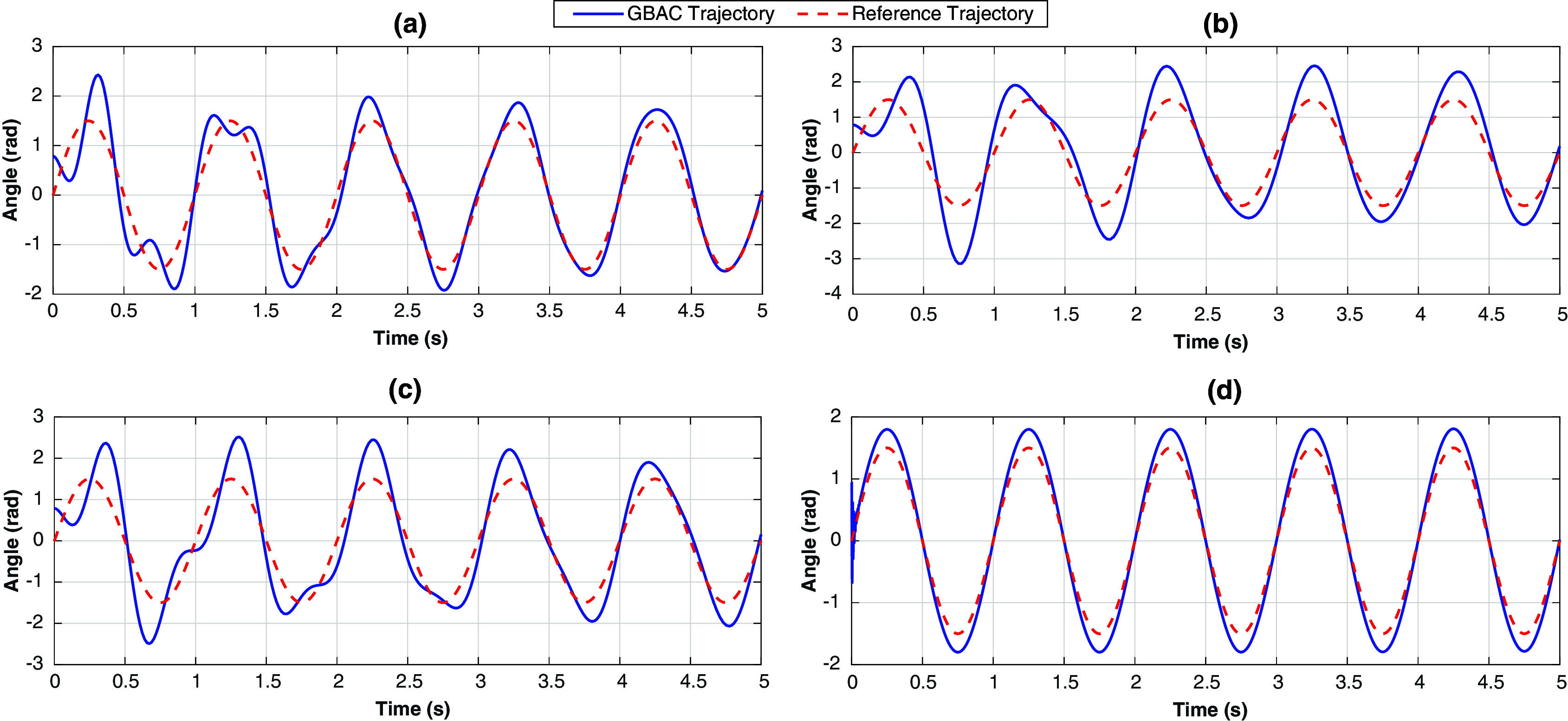
Simulation of a 1-DOF exoskeleton tracking reference trajectory (red dashed line) with the proposed Gaussian-based adaptive control controller (black line) with the following representative uncertainties. (a) Spring stiffness increased by 100% (nominal: 25 N/m). (b) Pendulum length increased by 50% (nominal: 0.35 m). (c) Damping coefficient increased by 100% (nominal: 0.08 N s/m). (d) Mass increased by 100% (nominal: 0.5 kg).

Figure 4a shows the effect of increasing the spring stiffness by 100% above the nominal value of 25 N/m. GBAC quickly adapts to this disturbance and closely tracks the reference trajectory with an overall RMS error of 0.12 rad across the simulation window (5 s). Figure 4b explores the influence of a 50% increase in pendulum length (nominal value 0.35 m), with a greater impact on system performance than spring stiffness (RMS = 0.17 rad). Figure 4c demonstrates the effect of doubling the damping coefficient on the system’s oscillatory behavior, with the largest impact of the simulated parameter changes (RMS = 0.42 rad). Figure 4d illustrates the relative robustness of GBAC to an unexpected increase in mass (100% of the nominal value of 0.5 kg), resulting in an RMS error of 0.19 rad. We also completed simulations to evaluate the Slotine–Li and PID controllers in the presence of spring stiffness and additional mass, and those results are discussed in comparison with the experimental study below. Collectively, these simulations demonstrate the GBAC controller’s robustness and adaptability to maintain suitable system tracking performance in the presence of unmodeled disturbances and significant parameter uncertainties.

### 4.2. Experimental study

**Figure 5.**
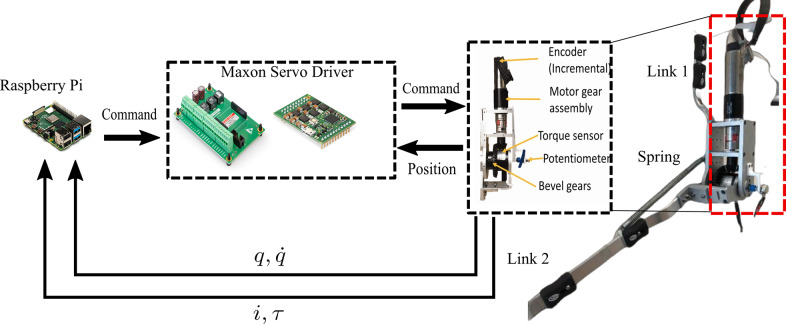
The 1-DOF knee exoskeleton used in the experimental study, including the Raspberry Pi for the implementation of the closed-loop control and the Maxon servo drivers for issuing motor commands.

Whereas the simulation study evaluated controller performance in 1-DOF and 2-DOF exoskeleton, our experimental evaluation focused on evaluating the adaptive control strategies in our existing 1-DOF knee exoskeleton, which was designed for gait assistance and rehabilitation in children with a limb length of 42–66 cm and a total limb mass of 0.8–3.4 kg who have knee extension deficiency from cerebral palsy and other movement disorders. The mechanical and electrical design details of the exoskeleton have been previously reported (Chen et al., 2021) and are summarized briefly here. The knee exoskeleton is a two-link mechanism actuated by a geared BLDC motor (Figure 5). The actuator is a high-precision motor (Maxon 323218) equipped with a two-stage gear reduction. The first stage is a planetary gearbox (Maxon 370782) with an 89:1 reduction ratio. The second stage is a right-angle bevel gearbox with a 3:1 reduction ratio.

Hall effect sensors and a 512 counts per turn digital encoder (Maxon MR 128–512) measure the rotor and shaft position (and speed), respectively. A potentiometer is attached to the motor shaft to calibrate the encoder. In this experimental setup, Link 1 has one side of a tension spring (25 N/m spring constant) attached 5 cm proximal to the knee joint, with the other side attached to Link 2, 15 cm distal to the joint. The spring was intended to represent unmodeled dynamics typically encountered during exoskeleton use, that is, the undesired muscle force created in response to exoskeleton assistance, possibly due to spasticity.

A servo control module (Escon 50/8) powered the knee exoskeleton actuator. The module has built-in PD current control capability and an accurate current measurement that implicitly represents the joint torque and power. The mechanical and electrical parameters of the 1-DOF knee exoskeleton and actuator are provided in Table 1.

**Table 1.** Electrical and mechanical parameters of the 1-DOF knee exoskeleton

Parameter	Value	Unit	Description
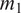	2.15	kg	Mass of Link 1 (thigh)
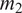	0.5	kg	Mass of Link 2 (shank)
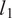	0.25	m	Length of Link 1
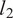	0.35	m	Length of Link 2
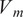	24	V	Motor’s nominal voltage
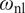	163,000	rpm	No load speed
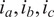	0.164	A	No load current
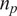	2		Number of pole pairs
*R*	0.527	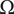	Phase resistance
*L*	50 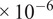	H	Phase inductance
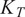	0.014	Nm/A	Torque/back-EMF constant
*J*	1.7 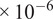	kg m^2^	Rotor inertia
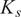	680	rpm/V	Speed constant
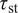	0.164	A	Stall torque
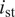	45.5	A	Stall current
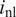	0.639	Nm	No load current

The adaptive control laws were implemented on a Raspberry Pi (model B) single-board computer (Figure 5). The Pi calculates the torque command for the exoskeleton (e.g., using equations (16) and (17) for the GBAC controller), which was then scaled as an analog control signal between 0 and 5 V and sent as an analog input to the Escon module to set the motor torque value. Like the simulation, five Gaussian regressor nodes were selected, with an initial value of 0 and initial values of 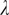 and 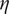 set to [10 10] and 75, respectively. The overall sampling time for the closed-loop adaptive controllers was 20 ms, including the measurement and calculation latency between the Escon module and the Raspberry Pi. However, for the PID controller, a higher sampling rate (12 ms) was used because of its less complex calculations. The PID gains were set as [43, 16, and 33] for P, I, and D gains, respectively. The recording time for the knee joint position and velocity on the Raspberry Pi SIM card contributed approximately 5-ms latency for every 500 samples. The reference trajectory was selected to be a sinusoidal signal with a frequency of 1 Hz and an amplitude of 0.785 rad (45 degrees). The tension spring of 25 N/m constant was utilized to form a flexible actuated joint and was set at an initial angular position of 45 degrees with the vertical axis. The joint angular position and velocity were recorded by the motor encoder during the testing session for each controller.

**Figure 6.**
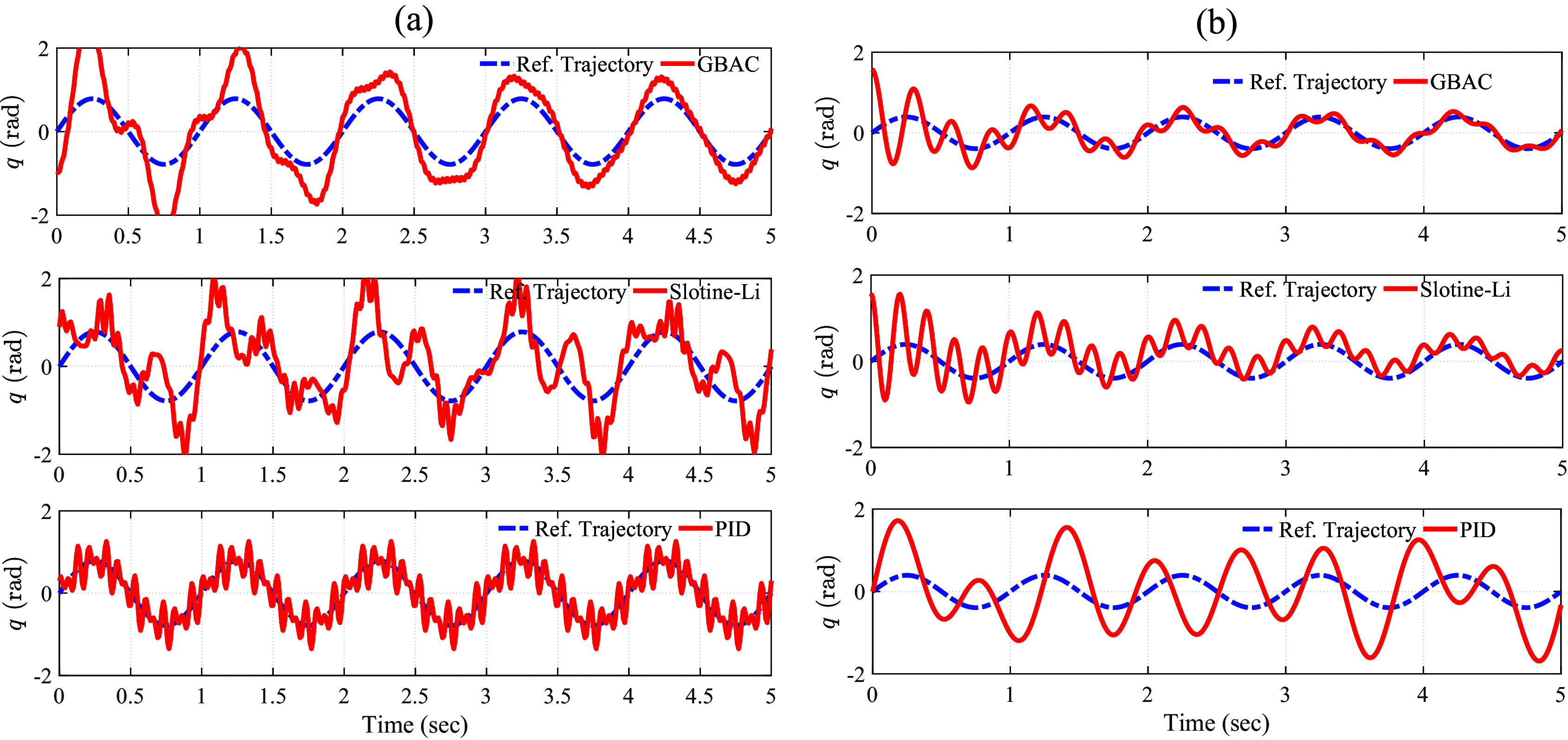
(a) Experimental results from the 1-DOF tracking task with a 25 N/m tension spring displayed as the measured knee angle (red) and the reference angle (blue) for the Gaussian-based adaptive control (top), Slotine–Li (middle), and PID (bottom) controllers. (b) Controller performance presented in the same format as (a) but with a 0.5-kg mass added to the shank center of mass. A low-pass filter with a 20-Hz cutoff frequency was used to smooth measured angular position and velocity.

Figure 6a shows the experimental study outcomes. The experimental results showed that the GBAC had an initial RMS error that was similar to the Slotine–Li and PID controllers; however, the GBAC quickly converged to a solution with a lower mean RMS error, eventually reaching 0.2 rad after 4 s. The Slotine–Li controller provided stable tracking but with a slower convergence rate than the GBAC control. The RMS error for the final 1 s was 0.64 rad. Finally, the PID controller showed stable but oscillatory tracking around the reference trajectory, with a consistent RMS error of 0.71 rad. The PID performance was attributed to the uncertainty due to the spring force, for which the PID controller is less effectively able to compensate compared with the adaptive control approaches. These experimental results align with those from the 1-DOF simulations. Collectively, the simulation results showed a lower RMS error than in the experiment (Table 2); however, in both the simulation and the experiment, the GBAC had the lowest RMS error, followed by Slotine–Li and then PID.

**Table 2.** Comparison of simulation and experimental results

	Simulation			Experimental		
	GBAC	SL	PID	GBAC	SL	PID
Spring RMS (rad)	0.17	0.36	0.4	0.2	0.64	0.71
Mass RMS (rad)	0.20	0.30	0.60	0.87	0.87	1.56

To further validate the GBAC performance in controlling a flexible joint (mechanism plus spring), a second experiment was performed with a 0.5-kg mass added to the shank center of mass. This makes the exerted external force vary between 4.9 N at the horizontal position of the shank and 2.45 N at the end of the range of motion. This additional mass was intended to simulate the weight of a limb that would not be present during initial system tuning.

The tracking performance of all controllers is clearly affected by the presence of the additional mass (Figure 6b). Both the GBAC and Slotine–Li controllers converge under this situation of high uncertainty (variable shank mass) and disturbance (spring flexibility), which constitute a considerable challenge for controllers without adaptation, such as the PID controller. Notably, the PID controller, as shown in Figure 6b, displayed the worst tracking performance with an RMS error value over the 5-s test period of 1.56 rad. On the other hand, GBAC and Slotine–Li were able to adapt and show convergence within 5 s. The Slotine–Li controller shows an oscillatory behavior during its convergence, resulting in an overall tracking RMS error of 0.87 rad. Similar oscillatory behavior was observed with GBAC during the first 2 s; however, GBAC overcomes this, likely due to the adaptation facilitated by the Gaussian nodes, as shown in equation (14). As in the first experiment, the results with additional mass followed the same pattern as the 1-DOF simulation, with RMS error being higher for all controllers in the experimental results (Table 2). Interestingly, the GBAC showed less RMS error than the Slotine–Li in simulation but similar in the experiment. In both cases, the PID controller had the worst tracking performance with the unexpected addition of mass to the flexible joint.

## 5. Discussion

In this work, motivated by the inherent control challenges of lower extremity exoskeletons with human users, we developed a Gaussian regressor-based approach to adaptive control for flexible exoskeleton joints. The Gaussian regressor approach has previously been shown to be capable of adaptively compensating for uncertainty in system dynamics to achieve stable and accurate tracking control (Sanner and Slotine, 1992). Here, we derived the system dynamics of a lower-limb exoskeleton using Lagrangian mechanics and applied a Gaussian regressor to approximate the system uncertainties and update the system dynamics at each time step. This formulation does not constrain the updated control parameters to a Lyapunov stability region and thus, provides a potential operational advantage in adapting the feedback gains to minimize tracking error in the presence of uncertain system dynamics. Further, this approach offers the benefit of rapid convergence with limited and/or noisy data and when the overall system dynamics may not be well characterized, all of which make it attractive for control of pediatric exoskeletons. Yet, the GBAC technique utilizes a statistically based kernel to update the control parameters, which can result in slower convergence if the uncertainty is not well captured. Also, GBAC does not take advantage of the data itself to improve controller performance over time. In the future, Gaussian regression-based control could be combined with other techniques to further improve adaptive control. For example, Gaussian regression-based control could be deployed to generate a set of initial control inputs, and then reinforcement learning could be deployed to improve the control policy over time.

Our simulation and experimental results validate the effectiveness of the proposed GBAC technique in 2-DOF and 1-DOF tracking tasks, respectively. In the simulation of tracking anatomical hip joint trajectories with relatively low uncertainty, GBAC and Slotine–Li adaptive controllers, as well as PID, performed well. This result was expected due to the relatively low frequency of hip motion during walking and its relatively high inertia. When uncertainty was introduced through increased mass and length, the adaptive controllers converged to an effective solution compared to the PID. At the knee, which poses a more difficult tracking task, all three adaptive controllers significantly outperformed the PID controller (RMS error = 1.21) when the system was simulated at 5% above the modeled mass and length. However, when uncertainty was elevated to 100% of system mass and length, the GBAC and BS outperformed Slotine–Li adaptive control, and while both had similar RMS error during the first 2 s of the task, GBAC did converge faster in this case (Figure 2). Further, we observed that the GBAC Gaussian nodes demonstrate consistent convergence with stable values in less than 2 s. Simulated and experimental results also show effective control with GBAC in a single joint system. In the simulated response to an external disturbance, GBAC showed superior robustness, quantified by RMS error, compared with the Slotine–Li and PID controllers (Figure 3). Simulations further demonstrated the ability of GBAC to converge to accurate trajectory tracking control, despite high parameter uncertainty in the form of increased spring stiffness, damping coefficient, link length, and mass (Figure 4). The simulated 1-DOF results also showed improved performance of GBAC compared with the Slotine–Li and PID control approaches in response to unmodeled changes in system dynamics induced by a tension spring and added mass (Table 2). And while the tracking results in the experiment were higher than those in the simulation for all controllers, GBAC achieved an experimental tracking error of less than 0.2 rad after 4 s in our exoskeleton setup, lower than the other controllers (Figure 6).

There are several limitations to the existing study that warrant discussion. First, our simulation and experimental results included system uncertainties in link mass, length, and external dynamics represented by a spring. While these represent real challenges to exoskeleton control, for example, across a range of individuals and in the presence of undesired external forces such as those generated by the user’s muscles, they do not encompass all sources of potential uncertainty. Therefore, continued investigation of GBAC performance in real-world exoskeleton applications is needed. We also note that the experimental performance of the GBAC was limited by the hardware implementation. Specifically, expanding GBAC beyond five Gaussian nodes is theoretically possible, but the Raspberry Pi was unable to compute the updates at a sufficient speed beyond this number. This can be addressed by improving the hardware used for real-time control. We are also investigating the use of non-homogenous Gaussian nodes to improve convergence.

The next steps in this work will be to evaluate GBAC stability in simulation and in single- and multi-DOF exoskeletons, as well as its performance in tracking more complex movement trajectories. Ultimately, the objective is to deploy GBAC as a data-driven controller for lower-limb exoskeleton use across multiple individuals and activities with minimal *a priori* tuning. Future work will include its evaluation in this context in comparison with other state-of-the-art methods such as BS control. We anticipate the first application in our existing pediatric knee exoskeleton for overground walking, although it is possible to extend this approach to multi-joint exoskeleton control.

## Data Availability

Data and associated code from this study are available from the corresponding author by email request.

## Appendix: GBAC stability

To prove GBAC stability, we define a Lyapunov function candidate  as

(A1)

This function is positive-definite, as  and  for all  and  , and  if and only if  and  . The Lyapunov function  encapsulates both the tracking error  and the weight estimation error  , providing a measure of the total energy of the system. The time derivative of  is computed as

(A2)

From the system dynamics, the filtered error  is defined as

(A3)

Substituting this into the expression for  , we obtain

(A4)

Substituting the update law from equation (22) into the expression for  , we derive

(A5)

Simplifying this expression yields

(A6)

The term  represents the effect of the system uncertainties on the tracking error. From the system dynamics, the tracking error is governed by

(A7)

Using the definition of the filtered error  , we can rewrite the tracking error dynamics as

(A8)

This relationship establishes a direct connection between the weight estimation error  and the filtered error  . Substituting  into the expression for  , we obtain

(A9)

Expanding and simplifying this expression yields

(A10)

To analyze the sign of  , we observe the following:

- The term  is bounded by  using the inequality  .
- The terms  and  are negative-definite.
- The term  is also negative-definite, as  is proportional to  .

Thus,  can be bounded as

(A11)

This inequality demonstrates that  is negative-definite. Since  is negative-definite, the Lyapunov function  decreases monotonically over time. This implies the following:

- The tracking error  , as  .
- The weight estimation error  , as  .

Therefore, the update law in equation (22) ensures *asymptotic stability* of the closed-loop system. The system is guaranteed to converge to the desired trajectory, and the Gaussian network weights adapt to compensate for the system uncertainties.
